# Soil contamination with silver nanoparticles reduces Bishop pine growth and ectomycorrhizal diversity on pine roots

**DOI:** 10.1007/s11051-015-3246-4

**Published:** 2015-11-21

**Authors:** M. J. Sweet, I. Singleton

**Affiliations:** Environmental Sustainability Research Centre, College of Life and Natural Sciences, University of Derby, Kedleston Road, Derby, DE22 1GB UK; School of Biology, Newcastle University, Ridley Building, Newcastle upon Tyne, NE1 7RU UK; School of Life, Sport and Social Sciences, Edinburgh Napier University, Sighthill campus Sighthill Court, Edinburgh, EH11 4BN UK

**Keywords:** AgNP, Fungi, Pine, Nanoparticle, Environmental effects

## Abstract

Soil contamination by silver nanoparticles (AgNP) is of potential environmental concern but little work has been carried out on the effect of such contamination on ectomycorrhizal fungi (EMF). EMF are essential to forest ecosystem functions as they are known to enhance growth of trees by nutrient transfer. In this study, soil was experimentally contaminated with AgNP (0, 350 and 790 mg Ag/kg) and planted with Bishop pine seedlings. The effect of AgNP was subsequently measured, assessing variation in pine growth and ectomycorrhizal diversity associated with the root system. After only 1 month, the highest AgNP level had significantly reduced the root length of pine seedlings, which in turn had a small effect on above ground plant biomass. However, after 4 months growth, both AgNP levels utilised had significantly reduced both pine root and shoot biomass. For example, even the lower levels of AgNP (350 mg Ag/kg) soil, reduced fresh root biomass by approximately 57 %. The root systems of the plants grown in AgNP-contaminated soils lacked the lateral and fine root development seen in the control plants (no AgNP). Although, only five different genera of EMF were found on roots of the control plants, only one genus *Laccaria* was found on roots of plants grown in soil containing 350 mg AgNP/kg. At the higher levels of AgNP contamination, no EMF were observed. Furthermore, extractable silver was found in soils containing AgNP, indicating potential dissolution of silver ions (Ag+) from the solid AgNP.

**Electronic supplementary material:**

The online version of this article (doi:10.1007/s11051-015-3246-4) contains supplementary material, which is available to authorised users.

## Introduction

Nanoparticles are increasingly being used in a wide variety of commercial applications, and this widespread use means that they will inevitably become common environmental contaminants. This contamination can occur either, indirectly, by entering waste streams for example, or directly, in the case of agricultural applications (Zhang et al. 2012). Silver nanoparticles (AgNP) in particular are used extensively due to their antimicrobial properties (Marambio-Jones and Hoek 2010; Mathew and Kuriakose 2013), and AgNPs are currently utilised commercially in such instances as textiles, disinfectants, chopping boards, washing machines and even for organ transplantation (Sweet and Singleton 2011). Recent work has shown that AgNP-treated commercial clothing (e.g. socks and t-shirts) can release a significant amount of AgNP into the environment via the water from washing machines (up to 650 mg/500 mL water). This provides a pathway whereby AgNP can reach the external environment, via waste-water treatment plants and ultimately entry into sewage sludge/biosolids (Benn and Westerhoff 2008). Other authors have also highlighted the potential for nanoparticles to enter the environment from different consumer products (Benn et al. 2010; Farkas et al. 2011). Biosolids are often used in commercial forestry and can be used to enhance seedling establishment (Valdecantos and Cortina 2011). This direct use of contaminated biosolids means that young trees (such as young pine) and their associated microbes could be directly exposed to nanoparticles. Trees, such as pine, benefit from fungal associations with their roots (Sousa et al. 2012), and these ectomycorrhizal fungi are proposed to aid tree growth by various potential mechanisms including improved nutrient uptake and stress tolerance (Finlay 2008; Gordon and Gehring 2011). Soil contamination with AgNP has been shown to affect specific microbes; however, much of the work has been focused on effects on bacteria, such as species from the genus *Bradyrhizobium* (Kumar et al. 2011). As far as the authors are aware, no work has been carried out on the effect of AgNP contamination on beneficial ectomycorrhizal fungal colonisation of tree roots despite the known antifungal effects of AgNP (George et al. 2011; Jo et al. 2009; Min et al. 2009). In addition, it is possible that growth of the trees themselves could be directly affected by the presence of AgNP as plants are known to be sensitive to nanoparticles (Yin et al. 2012). Therefore, this study aimed to determine the effect of AgNP contamination of soil on: (a) pine tree growth rates (shoot and root growth) and (b) ectomycorrhizal fungal colonisation of the pine tree roots.

## Experimental

### Soil preparation

Soil (the top 10 cm below the easily removed litter layer) was collected from a forested area of Point Reyes National Seashore (PRNS), California, USA, see (Branco et al. 2013) for site details. GPS location: N38 05.087 W122 52.253. After the removal of stones and larger material, the soil was air dried for 48 h prior to being sieved to 2 mm in the laboratory. Sterile sand (autoclaved for 30 min on three successive days) was added to the soil to 30 % v/v to improve aeration during the experiment. AgNP (20 nm diameter, 99.8 % purity, obtained from US Research Nanomaterials Inc, Texas 77084, USA) were added to a smaller portion of the soil (~100 g) and mixed thoroughly (for 10 min using a metal spatula) to obtain a homogenous dispersion of AgNP. This 100 g of soil was then thoroughly mixed into larger soil volume in ‘zip-loc’ bags to obtain final AgNP levels of 350 and 790 mg Ag/kg (see below). These AgNP levels were chosen as they were similar to those used in previous work (Kumar et al. 2011) and represent a high level of AgNP contamination. Non-contaminated control soil was also prepared in the same way but without the addition of AgNP. The soil:sand mix (65 ml volume) was then added to individual ‘cone-tainers’ (Steuwe and Sons, Corvallis, USA) and covered with a 1 cm depth of sterile sand. Altogether 14 replicates of each treatment (0, 350 and 790 mg Ag/kg) were prepared.

### Soil analysis

Dried soil (40 °C) was analysed by the UC Davis College of Agricultural and Environmental Sciences Analytical Laboratory using standard methods (prior to experimental set-up). Soil texture pH, organic C, total N, total P (Olsen), total silver and extractable silver were determined and results reported in Tables 1, 2 and 3.

**Table 1:** Soil properties

Total organic carbon (%)	3.88
Total N (%)	0.31
Olsen-P (mg/kg)	15.50
pH	4.94
Sand (%)	58.00
Silt (%)	18.00
Clay (%)	24.00

**Table 2:** Ectomycorrhizal genera fund on roots from soils containing 0, 350 and 790 mg Ag/kg

Control	350 mg AgNP/kg	750 mg AgNP/kg
*Laccaria* (×3)	*Laccaria*	None found
*Thelephora*		
*Rhizopogon occidentalis* (×2)		
*Tomentella* (×2)		
*Tuber*

**Table 3:** Total and extractable Ag levels in contaminated soil samples

Total Ag in soil (mg/kg)	Extractable Ag in soil (mg/kg)
Control	<0.01 (below detection limit)
350	12.07 ± 0.85
790	15.44 ± 1.19

#### Analysis of total silver in soil

Soil samples were digested by nitric acid/hydrogen peroxide closed vessel microwave digestion and the total amount of silver in the digest analysed by ICP-AES (UC Davis standard method 590.02).

#### Extractable silver analysis of soil

The level of extractable silver in triplicate samples obtained from each treatment at the end of the plant growth period (4 months: see below) was determined by the method of Hou et al. (2005). Briefly 1 g soil was added to 10 ml of 1 M NH_4_NO_3_ (pH 7) and shaken at 100 rpm in an orbital shaker for 4 h at 25 °C. The extract was collected by centrifugation at 3000 rpm×g for 10 min. Extracts were stored at −20 °C until analysis by ICP-AES using standard methods at UC Davis.

### Preparation and growth of *Pinus muricata* D. Don (Bishop pine) seedlings

*Pinus muricata* cones were collected from different trees in PRNS and dried in the laboratory to allow collection of seeds. Wings were removed from seeds and stored at 4 °C until required. To start germination, seeds were placed in 15 % (v/v) H_2_O_2_ solution plus tween 80 (one drop per 500 ml) and stirred for 15 min. Seeds were then collected in a sieve, rinsed with deionised water and finally soaked in deionised water for 24 h prior to planting in soil. Three seeds were planted in each cone-tainer (prepared as described above) and distilled water added until saturated soil moisture conditions were achieved (maintained throughout the experiment). Cone-tainers were incubated at 20 °C in a growth chamber set at a constant light intensity of ~220 µmol m^−2^s^−1^.

### Sampling of plants and soil

Seedlings were thinned to one per cone-tainer after a period of 1 month, and the thinned seedlings used for initial experimental observations of root length, root and shoot fresh weight. The remaining seedlings were grown for a further 4 months and destructively harvested for measurement of shoot and root fresh weight and ectomycorrhizal diversity on roots. Soil was also analysed for extractable silver levels after 4 months (see above).

### Collection of ectomycorrhizal roots, DNA extraction and PCR

 Root tips were collected from a random subsample (from five cone-tainers) of the different AgNP-treated pine seedlings. The aim of the experiment was to observe the total diversity of ECM present. So roots that displayed different ectomycorrhizal root morphology (such as variations in colour, diameter and tissue density (Comas et al. 2014) were preferentially collected. Most of the AgNP-treated plants showed no obvious visual ECM colonisation so ‘normal’ roots were collected in an attempt to discover if any ectomycorrhizal colonisation was present. Overall, a total of 10 root tip samples were collected from each treatment and were subjected to immediate extraction using the REDExtract-N-Amp Tissue PCR Kit (Sigma-Aldrich, Saint Louis, MO, USA). Each root tip was added to 20 μL of extraction buffer and incubated at 95 °C for 10 min. Then 20 μL neutralisation buffer was immediately added and the extracts stored at −20 °C prior to PCR. PCR was carried under using standard conditions with the fungal specific primer pair ITS1f and ITS4 (Gardes and Bruns 1993; White et al. 1990). PCR products were cleaned using AmPURE magnetic beads following manufacturers recommendations. PCR products were sequenced in forward and reverse directions using an ABI3170 Genetic Analyser (Applied Biosystems, Foster City, CA, USA). Fungi were defined using a 97 % sequence similarity cut-off and named according to the nearest BLAST match.

### Statistical analysis

All data were analysed by one-way ANOVA and differences between individual means were determined by post hoc least significance difference analysis using SPSS version 21.

## Results

**Fig. 1:**
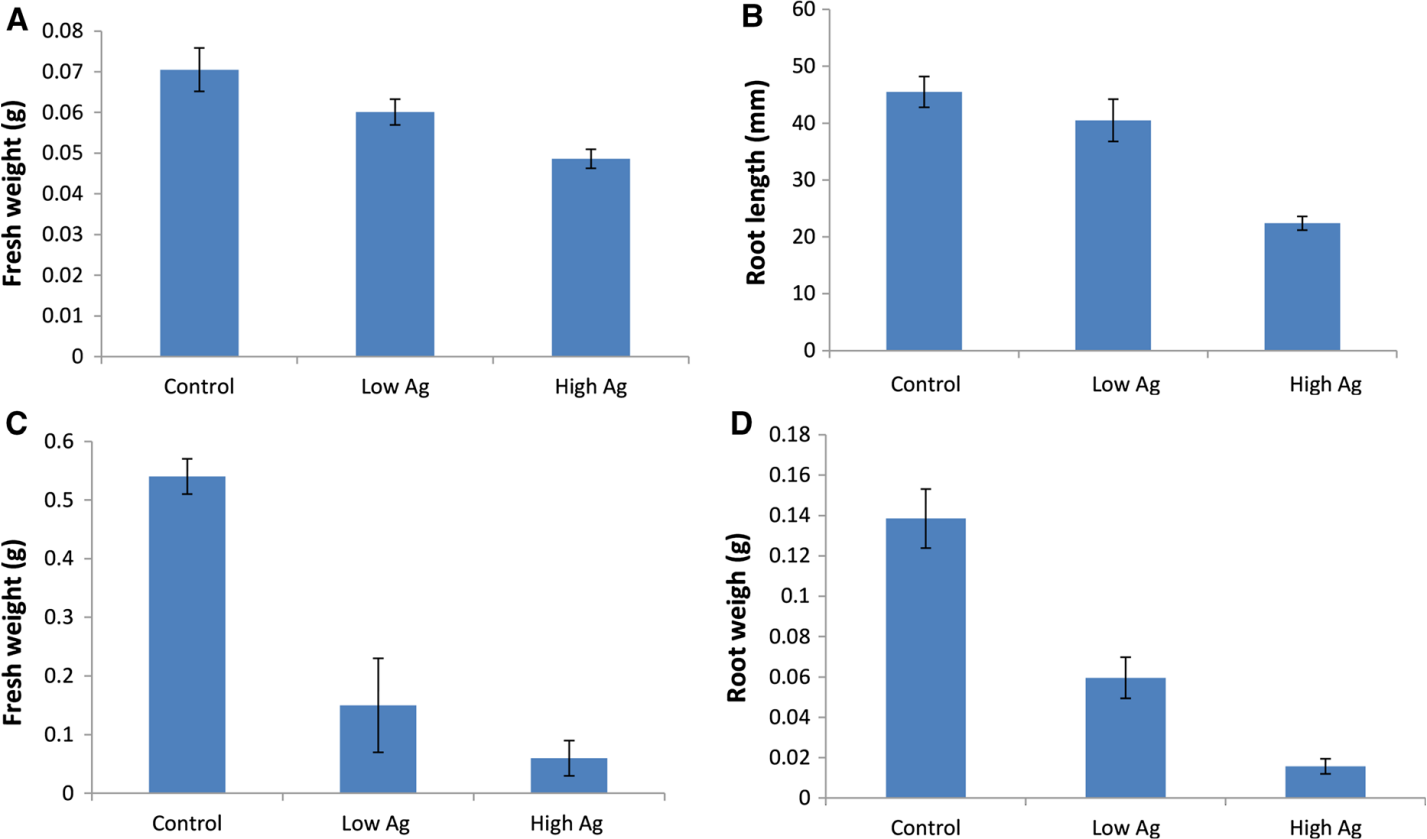
**A** Illustrates Bishop pine shoot fresh weight after one month of growth in soil containing different levels of AgNP. **B** The effect of AgNP soil contamination on Bishop pine root length after 1 month of growth. **C** The reduction in shoot fresh weight caused by AgNP soil contamination after 4 months growth and **D** reduction in root fresh weight caused by AgNP after 4 months growth. Control related to 0 mg Ag/kg, low levels relate to 350 mg Ag/kg and high related to 790 mg Ag/kg

No effect of AgNP contamination was observed on seedling germination and emergence (results not shown) and subsequently tree growth was analysed after 1 and 4 months. After 1 month, shoot fresh weight in the highest Ag level was slightly but significantly (*p* < 0.05) reduced by approximately 15 % (Fig. 1A) compared to the non-contaminated control, while shoot fresh weight at the lower AgNP level was not significantly affected. The primary tap root produced by pine in the presence of higher AgNP levels was significantly shorter (*p* < 0.05) than the primary roots produced in control and lower AgNP levels (Fig. 1B, supplementary Fig. 1) but had the same fresh weight value (data not shown) despite being shorter (supplementary Fig. 1). This appeared to be related to root thickness being increased at the higher AgNP level. After 4 months, both root and shoot growth were highly reduced in soils containing AgNP. For example, at 350 mg Ag/kg, shoot and root fresh weight was reduced by approximately 72 and 57 %, respectively (Fig. 1C, D).

### Molecular-based identification of ECM

PCR products were obtained for all the control root tip DNA extracts (10/10) and nine of these gave successful DNA sequences. In contrast, only three out of the ten samples with 350 mg Ag/kg revealed positive PCR products and only one produced a successful DNA sequence. For the final set of samples (790 mg Ag/kg), no PCR products were obtained for any of the root tip DNA extracts. Despite this, a random selection (n = 3) of these samples were still sequenced in case extremely low levels of PCR product were produced. Yet, no sequences were obtained for any of these samples.

Five ectomycorrhizal genera were found on roots of the control plants. Only one genus *Laccaria* was found on roots of pine grown in soil contaminated with 350 mg Ag/kg, and this was found on roots growing at the interface between the contaminated soil and sterile sand used to cover the soil surface. No ectomycorrhiza were found on roots in soil containing the highest AgNP level. Finally, we measured the levels of extractable silver in the soil samples. After 4 months, levels of extractable silver were determined to be approximately 3 % of the total silver present in the soil at 350 mg Ag/kg soil (Table 3). Extractable silver levels were found to increase in the soil containing more silver; howeve,r this was not significant.

## Discussion

The predicted increase in nanoparticle levels in sewage sludge and the applications of resulting biosolids to land (Judy et al. 2011) means that the effect of nanoparticle contamination on plant:microbial interactions requires further study. This work focussed on the effect of AgNP on establishment of ectomycorrhiza on Bishop pine. As far as we are aware, this is the first study of the effects of AgNP on pine growth and their ectomycorrhizal associations.

AgNP have varying effects on plants depending on the plant species, growth conditions (e.g. growth in soil or different nutrient media) and the level and type of AgNP applied (e.g. surface modified or untreated) making result comparisons difficult. However, most studies have shown that AgNP exposure of plants has a deleterious effect on growth. In this study, we show that pine seedling germination was not reduced by AgNP exposure while in contrast previous work has shown a variety of effects ranging from stimulation to a reduction in germination (Yin et al. 2012).

Here we show that root development in AgNP-exposed Bishop pine grown in soil was severely affected. Lateral root development was observed in controls (no AgNP) but lateral root formation in test treatments was reduced. In the highest AgNP level, only one vertical root was generally formed and only a few lateral roots were found in plants grown at the lower AgNP level (350 mg AgNP/kg), a result likely due to the roots being restricted to the soil surface layer (between the AgNP-contaminated soil and sterile sand added to the soil surface). Supporting these results, significant effects of AgNP on plant roots have been observed in previous studies on *Phaseolus radiatus* and *Sorghum bicolor* (Lee et al. 2012) and on wetland plants (Yin et al. 2012). Specifically, it has been suggested that AgNP exposure affects fundamental root growth processes such as gravitropism (Yin et al. 2011). It may be expected that the reduction in plant root growth caused by AgNP may lead to a reduction in above ground biomass, due to decreased nutrient uptake. This study showed exactly that, with contaminated soils showing lower levels of biomass. However, it may be possible that this reduced growth may be due to Ag+ or AgNP being taken up by the plant and translocated to the shoots, resulting in direct above ground toxicity effects. Indeed, plant uptake of gold nanoparticles has been observed in tobacco to the same effect (Judy et al. 2011).

The marked effect of AgNP exposure on plant roots (in particular less lateral roots formed) is the most likely explanation for the reduction in ectomycorrhizal diversity observed in this work. Ectomycorrhizal associations on roots from control soils were clearly visible and a variety of types were observed. However, no obvious ectomycorrhizal roots were seen in any of the AgNP-contaminated soils, and the few root tip samples available in silver exposed soils were taken in case any ectomycorrhizal root associations had formed but were not observable. The ectomycorrhizal species found in control soils were typical of those found previously in Point Reyes soils (Peay et al. 2010) and BLAST searches came up with matches most similar to ectomycorrhiza previously found in Point Reyes soil samples. The development of an ectomycorrhizal association with a plant root is a complex process with the precolonisation stage involving interactions between the plant host and the fungus (Ditengou et al. 2000; Felten et al. 2009; Martin et al. 2001) and mycorrhiza helper bacteria (MHB) (Bending 2007; Cusano et al. 2011). It is possible that AgNP exposure of plant roots, the fungal partner and MHB could affect such interactions thereby reducing the potential for mycorrhizal formation. Following root colonisation, it has been demonstrated that both fungal and plant gene and protein production alters in response to infection (Heller et al. 2008; Tarkka et al. 2001). Therefore, even if a fungus was able to initiate infection then the silver contamination may alter gene expression in both partners resulting in a reduction in speed or extent of colonisation.

Previous work examining the toxicity of silver and AgNP towards fungi has shown that AgNP levels below 10 ppm in agar can reduce fungal colony formation from conidia (Jo et al. 2009). Interestingly, the soil extraction technique used in this study indicated that a significant proportion of silver was available (12 mg Ag/L soil solution) and could therefore affect fungal growth assuming that the Ag present was in a form bioavailable to fungi. The toxic effect of silver on fungal conidial germination and growth would serve to reduce ectomycorrhizal root formation as fungal colonisation of roots from new seedlings would mainly be established via fungal spores from the existing soil spore bank or hyphal growth from an established symbiosis.

It is thought that the toxicity of AgNP is related to release of soluble Ag+ from the particle (Sweet and Singleton 2011) although there is evidence indicating that AgNP themselves can be taken up by cells (not observed yet with fungi) and release Ag+ intracellularly (Park et al. 2010). Fungal interaction with insoluble particles has been demonstrated previously (Singleton et al. 1990), so it is possible that AgNP could attach to fungal cell surfaces (spores and/or hyphae) and thereby deliver a concentrated pulse of Ag+ causing cell wall damage, preventing spore germination and/or reducing hyphal growth.

The extractability and toxicity of AgNP in soil is known to be dependent on a variety of soil factors (Calder et al. 2012; Coutris et al. 2012) and the availability of Ag from AgNP has recently been shown to increase with time using sequential extraction techniques (Coutris et al. 2012). Interestingly, both humic acids and microbes have been shown to cause AgNP formation from Ag+ (Akaighe et al. 2011; Sweet and Singleton 2011) which would theoretically reduce Ag bioavailability. Together, this presents a complex picture of AgNP behaviour in soil meaning that different soils will demonstrate different levels of Ag bioavailability and toxicity. It is also likely that plants and fungi will demonstrate differential access to the bioavailable fraction of Ag due to their varying abilities to take up Ag when complexed with soil derived compounds.

## Conclusions

Overall, AgNP contamination of soil resulted in a marked effect on Bishop pine root and shoot biomass and a reduction in ectomycorrhizal fungal species found in symbiosis with plant roots. It is likely that a combination of Ag derived toxicity effects on plant roots and fungal symbionts reduced the diversity of ectomycorrhizal fungi found. The levels of AgNP used in this work were relatively high, and it is recommended that future work be carried out with a range of AgNP levels. We propose that lower levels of AgNP could still affect ectomycorrhizal symbiosis due to the subtle interactions occurring between the plant host, fungal symbiont, and MHB on a gene expression level. Due to the complex behaviour of AgNP in soils, it is likely that the AgNP effects observed here will vary widely in soils of different characteristics and a range of soils should be examined. Finally, any future research must take into account the type of AgNP used (unmodified AgNPs were used in this work) as chemical modification of NP is common and such changes to AgNP are known to affect their behaviour in soil (Coutris et al. 2012).

## Electronic supplementary material

Supplementary Fig. 1 The effects of AgNP on pine growth and root length (TIF 8030 kb)
