# Supplementary figures and images for: Soil contamination with silver nanoparticles reduces Bishop pine growth and ectomycorrhizal diversity on pine roots
Source: J Nanopart Res. 2015 Nov 21;17(11):448. doi: 10.1007/s11051-015-3246-4 (PMC4655001; doi:10.1007/s11051-015-3246-4)

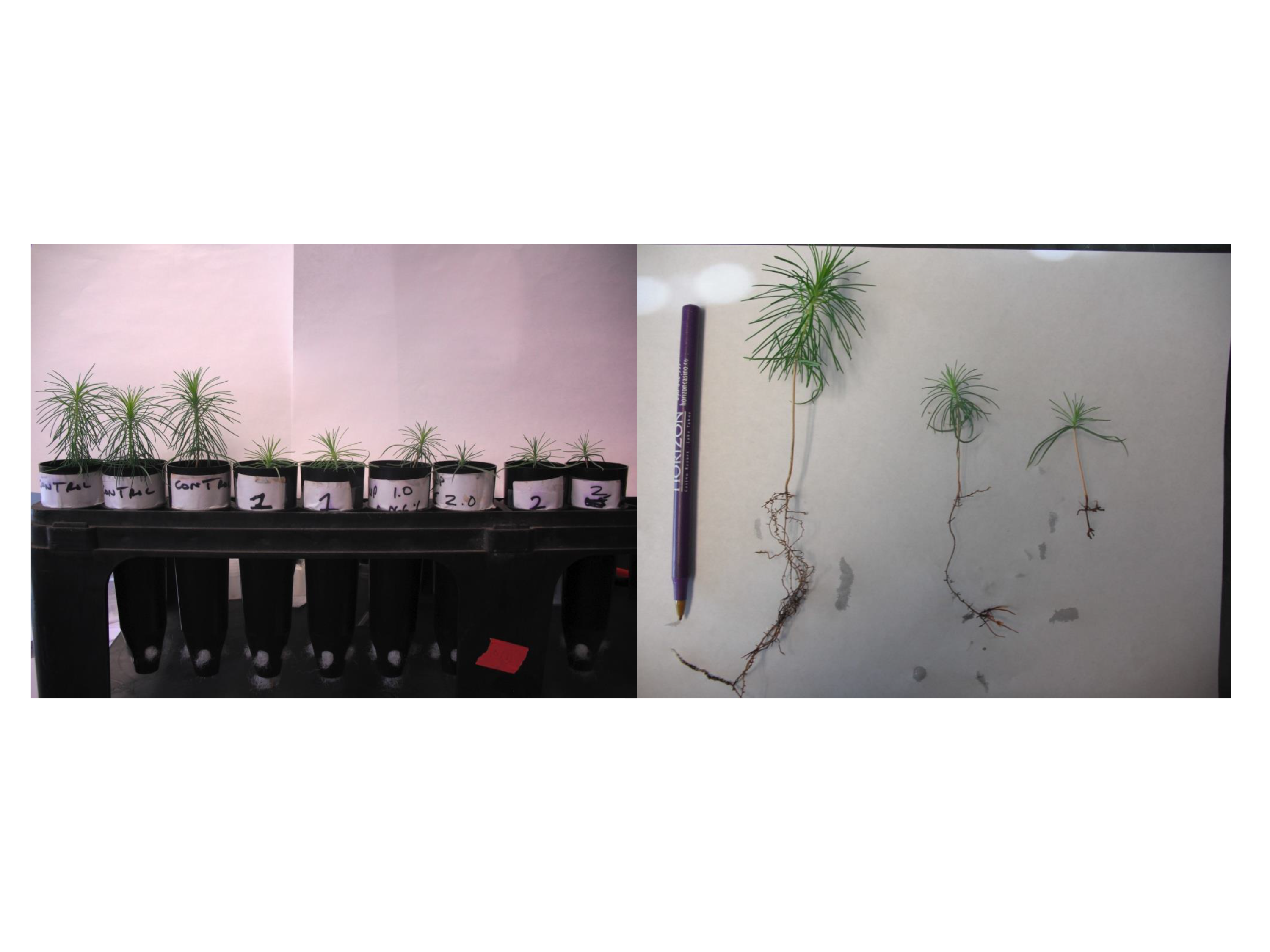

Supplement: Supplementary file 1 — Supplementary Fig. 1 The effects of AgNP on pine growth and root length (TIF 8030 kb) [file 11051_2015_3246_MOESM1_ESM.tif]
